# Arterial tortuosity syndrome with coronary artery involvement in a middle-aged female: A case report

**DOI:** 10.1016/j.radcr.2026.09.029

**Published:** 2026-10-10

**Authors:** Prinsa Shrestha, Amin Shah, Saroj GC, Niraj Kumar Sharma, Aditya Mahaseth, Sanjaya Kumar Shrestha

**Affiliations:** a Department of Internal Medicine, Kathmandu Medical College Teaching Hospital, Kathmandu, Nepal; b Department of Internal Medicine, Tribhuvan University Teaching Hospital, Kathmandu, Nepal; c Department of Radiodiagnosis and Imaging, Kathmandu Medical College Teaching Hospital, Kathmandu, Nepal; d Department of Cardiology, Kathmandu Medical College Teaching Hospital, Kathmandu, Nepal

**Keywords:** Arterial tortuosity syndrome, Coronary vessels, Elastic tissue, Vascular malformations, Tortuosity

## Abstract

Arterial tortuosity syndrome (ATS) is a rare autosomal recessive connective-tissue arteriopathy characterized by diffuse elongation and tortuosity of large and medium-sized arteries. Although it is typically diagnosed during childhood, adult presentation may occur and can be overlooked when overt connective-tissue manifestations are absent; coronary involvement is uncommon but clinically important. A 41-year-old woman presented with a two-week history of exertional substernal chest pain and dyspnea. CT coronary angiography demonstrated marked tortuosity of the left main coronary artery with focal kinking approximately 6.8 mm from its origin and 40–45% luminal stenosis (CAD-RADS 2). Contrast-enhanced CT angiography of the chest, abdomen, and pelvis demonstrated generalized arterial elongation and marked tortuosity involving the thoracic aorta, supra-aortic branches, and abdominal aortic branches, together with early branching of the main pulmonary artery, without aneurysm, dissection, or intramural hematoma. Conventional coronary angiography was technically unsuccessful because of severe arterial tortuosity, with unsuccessful catheter advancement and coronary engagement despite radial and subsequent femoral access. The overall clinical and imaging findings were highly suggestive of ATS with coronary involvement, although molecular genetic confirmation was not available. The patient was managed conservatively with vascular risk-factor optimization and structured follow-up, with symptomatic improvement at one month. This case highlights that ATS may present in adulthood with coronary artery tortuosity and focal kinking and emphasizes the importance of recognizing diffuse multi-territory arterial tortuosity on CT. Comprehensive vascular imaging can establish a strong radiological suspicion of ATS, identify coronary involvement and procedural challenges, and guide surveillance even when genetic confirmation is unavailable.

## Introduction

Arterial tortuosity syndrome (ATS) is an extremely rare autosomal recessive connective tissue disorder with an incidence < 1:1,000,000 live births, characterized by diffuse elongation, tortuosity and aneurysm of large and medium-sized arteries [1,2]. ATS is caused by biallelic pathogenic variants in SLC2A10, which encodes the facilitative glucose transporter GLUT10 [2]. Patients may present with features overlapping those of other connective-tissue disorders, such as Ehlers–Danlos and Marfan syndromes, exhibiting features such as joint laxity, hyperextensible skin, hernias, and cardiovascular manifestations; however, diffuse arterial tortuosity is a distinguishing feature of ATS [[1], [2], [3]].

Although ATS is classically presented in childhood, adult presentation with late or unexpected diagnoses has also been reported with or without skin and soft-tissue abnormalities [[4], [5], [6], [7], [8], [9]]. Coronary artery involvement is uncommon but clinically important and may pose diagnostic and procedural challenges [4,10]. Recognition of diffuse arterial tortuosity across multiple vascular territories is particularly important in adults, as the characteristic imaging phenotype may provide an important diagnostic clue even when overt connective-tissue features are absent [1,5,6,10]. Coronary involvement may further manifest as focal coronary artery kinking and can create technical challenges during conventional coronary angiography [4,10]. Here, we report ATS in a middle-aged woman presenting with exertional chest pain and dyspnea, with CT coronary angiography demonstrating left main coronary tortuosity with focal kinking and non-obstructive stenosis, alongside diffuse systemic arterial tortuosity.

This case report has been reported in line with the Case Report (CARE) guidelines [11].

## Case presentation

A 41-year-old women presented to our outpatient department with a two-week history of gradually progressive substernal chest pain and dyspnea on exertion. The chest pain was described as sudden onset, central, non-radiating, heaviness type, exacerbated by moderate exertion or stress. There were no pleuritic, sharp or positional components. The dyspnea began concurrently and gradually progressed, limiting her functional capacity to New York Heart Association (NYHA) class II. She denied orthopnea, paroxysmal nocturnal dyspnea, palpitations, syncope, dizziness, cough, fever, or lower-limb swelling.

Her past medical history was significant for chronic, relapsing symmetrical polyarthralgia involving both small (wrists) and large (knees) joints, described as intermittent and non-inflammatory, without associated swelling or warmth. She had undergone inguinal hernia repair with mesh placement 13 years earlier. The history of chronic polyarthralgia, although nonspecific, was considered in the context of the patient's broader connective-tissue phenotype, particularly in conjunction with the history of inguinal hernia and the characteristic diffuse arterial tortuosity identified on CTA. There was no history of diabetes mellitus and hypertension. There was no family history of connective tissue disorders or premature coronary artery disease and there was no parental consanguinity. She was a lifelong non-smoker, reported occasional alcohol consumption (1–2 standard drinks per week), and denied any illicit drug use. There were no known drug allergies (Table 1).

**Table 1:** Timeline of clinical events.

Time point	Clinical event
**Day 0 – presentation**	A 41-year-old woman presented with a 2-week history of exertional substernal chest pain and dyspnea (NYHA functional class II).
**Initial clinical evaluation**	Physical examination and initial investigations, including routine laboratory tests, electrocardiography, chest radiography, and transthoracic echocardiography, were performed. Echocardiography demonstrated preserved left ventricular systolic function (ejection fraction, 65%), mild tricuspid regurgitation, and no regional wall-motion abnormality.
**Attempted conventional coronary angiography**	Conventional coronary angiography was attempted for evaluation of suspected coronary artery disease. Radial access was initially unsuccessful, followed by femoral access; however, marked arterial tortuosity prevented adequate catheter advancement and selective coronary engagement despite multiple attempts.
**CT coronary angiography**	CT coronary angiography was subsequently performed and demonstrated marked tortuosity and focal kinking of the left main coronary artery approximately 6.8 mm from its origin, producing 40–45% luminal narrowing (CAD-RADS 2). The coronary calcium score was 0.
**Systemic vascular assessment**	Contrast-enhanced CT angiography of the chest, abdomen, and pelvis demonstrated diffuse arterial elongation and marked tortuosity involving the thoracic aorta, supra-aortic branches, and abdominal aortic branches. Early branching of the main pulmonary artery was also noted. No aneurysm, dissection, or intramural hematoma was identified.
**Radiological diagnosis**	The combination of diffuse arterial tortuosity and elongation with left main coronary artery kinking was considered highly suggestive of arterial tortuosity syndrome with coronary artery involvement. Genetic confirmation was not performed.
**Management**	The patient was managed conservatively with medical therapy and vascular risk-factor modification, with planned clinical and imaging surveillance.
**1-month follow-up**	At 1-month follow-up, the patient reported significant improvement in symptoms and remained clinically stable. Continued clinical and imaging follow-up was advised.

On physical examination, vitals were stable. She appeared comfortable at rest but experienced mild distress with movement. Cardiovascular examination revealed a regular rhythm with no murmurs, rubs, or gallops. Jugular venous pressure was not elevated, and peripheral pulses were symmetric. Respiratory examination showed clear lung fields with equal air entry. No lower-extremity oedema was present. The chest wall was non-tender, and skin examination revealed no pallor or cyanosis.

Initial investigations, including complete blood count, renal and liver function tests, thyroid function tests, urine routine examination, and serological studies, were within normal limits. The electrocardiogram showed a normal sinus rhythm with non-specific ST-segment and T-wave changes. Transthoracic echocardiography showed a dilated left atrium with preserved left ventricular systolic function (ejection fraction 65%) and mild tricuspid regurgitation (TRPG 25 mmHg) without any regional wall motion abnormality. Chest radiography demonstrated clear lung fields and widening of the mediastinum without other acute cardiopulmonary abnormalities.

Her basic investigations were within normal limits, so she underwent evaluation for suspected coronary artery disease. However, conventional coronary angiography (CAG) was technically challenging due to marked arterial tortuosity. Initial radial access was unsuccessful, necessitating conversion to femoral access; however, catheter manipulation remained difficult owing to significant tortuosity of the peripheral arteries and thoracoabdominal aorta, resulting in unsuccessful catheter advancement and coronary engagement despite multiple attempts (Fig. 1). Therefore, further evaluation with CT coronary angiography (CT-CAG) was performed, which demonstrated a calcium score of 0 with right-dominant coronary circulation and no anomalous coronary origin. CT coronary angiography demonstrated approximately 40–45% luminal stenosis of the tortuous left main coronary artery, with focal kinking approximately 6.8 mm from its origin, corresponding to CAD-RADS 2 (mild, 25–49% stenosis).

**Fig. 1:**
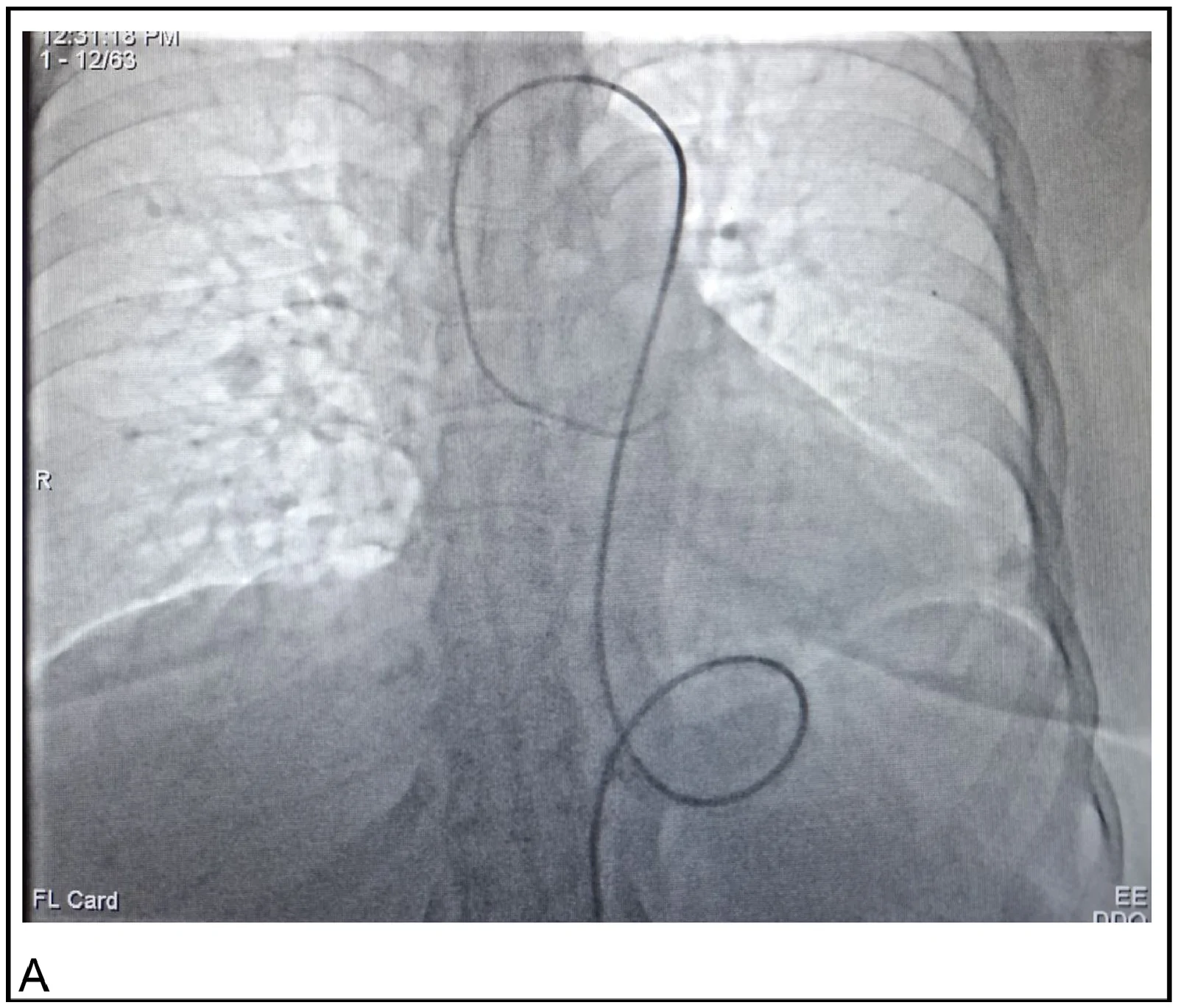
Fluoroscopic image obtained during attempted coronary angiography demonstrating severe arterial tortuosity of the thoracoabdominal aorta.

Contrast-enhanced CT angiography (CTA) of the chest, abdomen and pelvis with multiplanar and three-dimensional reconstructions demonstrated generalized arterial elongation and marked tortuosity. The major thoracic veins were patent without significant dilatation, thrombosis, or compression. Early branching of the main pulmonary artery was demonstrated on coronal reformatted images (Fig. 2A). Sagittal reformats showed marked elongation and tortuosity of the thoracic aorta, most prominent across the arch and descending thoracic aorta, without evidence of aneurysmal dilatation, dissection, intramural hematoma or mural thrombus (Fig. 2B). Volume-rendered reconstruction provided three-dimensional orientation of the pulmonary trunk branching pattern and the great vessels (Fig. 2C). Orthogonal CT aortographic measurements were within non-aneurysmal range for the imaged aortic segments, supporting tortuosity without aneurysmal dilatation.

**Fig. 2:**
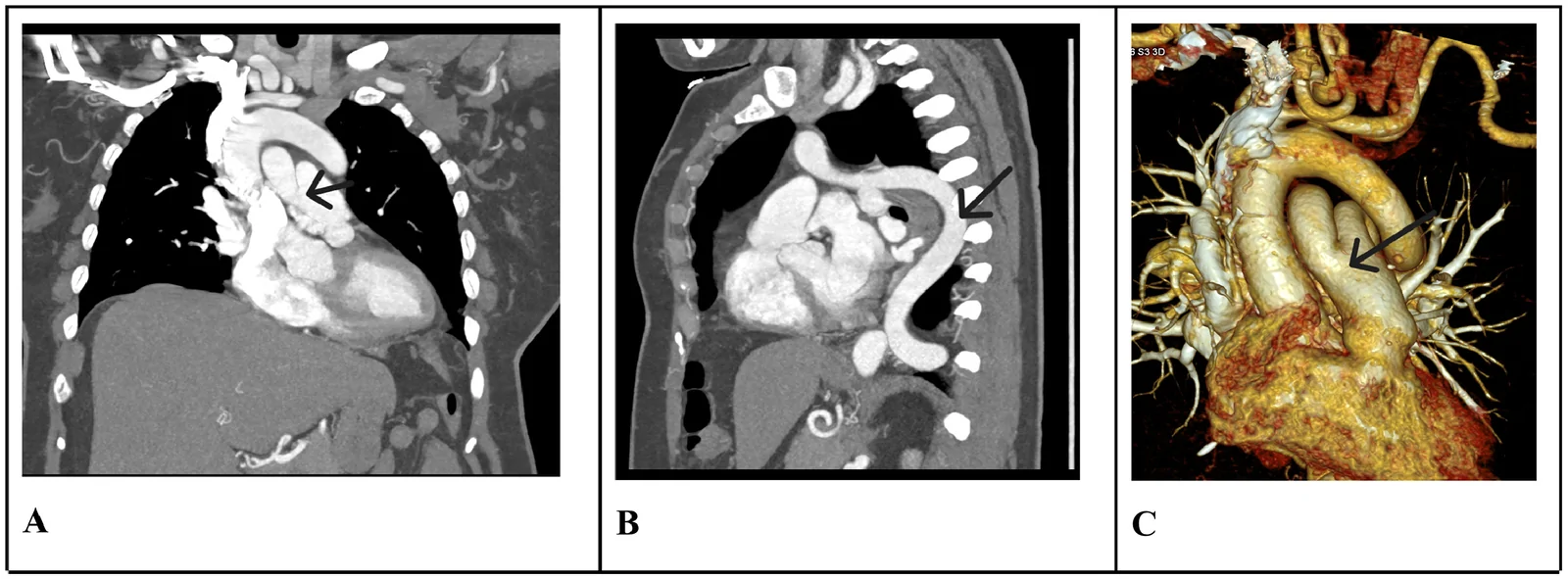
CT angiography Demonstrating early pulmonary trunk branching and marked tortuosity of the thoracic aorta. (A) Coronal reformatted image showing early branching of the main pulmonary artery (arrow). (B) Sagittal reformatted image demonstrating marked elongation and tortuosity of the thoracic aorta, particularly involving the aortic arch and descending thoracic aorta (arrow). (C) Volume-rendered reconstruction provided three-dimensional orientation of the pulmonary trunk branching pattern and the great vessels (arrow).

Reformatted CTA images at the thoracic inlet demonstrated tortuosity and looping of the supra-aortic branch vessels. A coronal reformat showed a looped course of a common carotid artery (Fig. 3A), with an additional vascular loop evident on axial imaging at a similar level (Fig. 3B). Three-dimensional volume-rendered reconstruction provided spatial orientation of the supra-aortic branches and illustrated the extent of branch-vessel tortuosity (Fig. 3C).

**Fig. 3:**
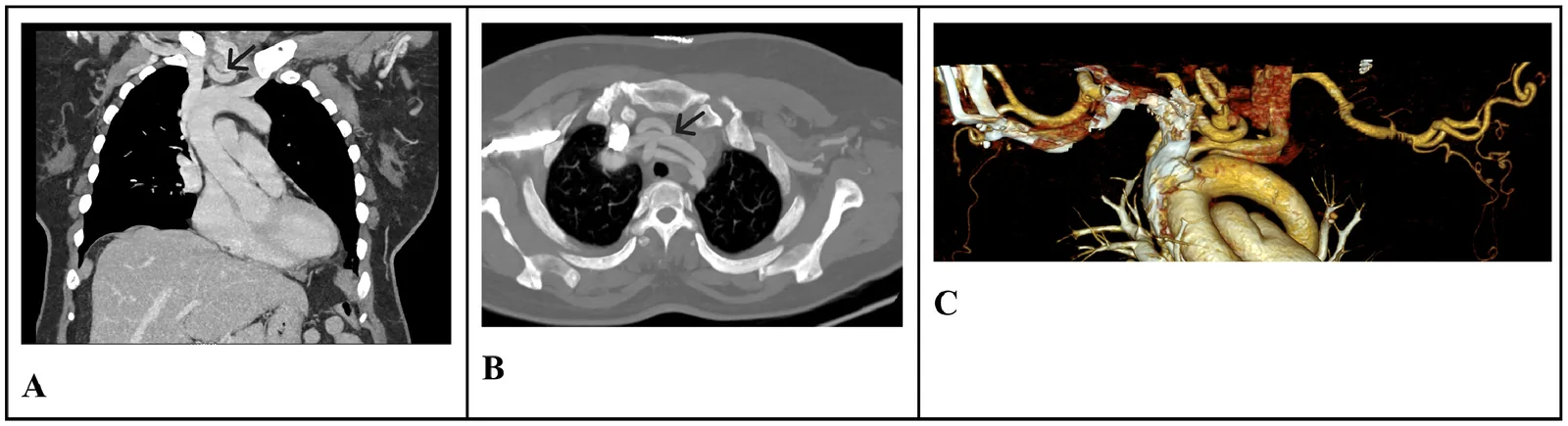
CT angiography demonstrating tortuosity and looping of the supra-aortic branch vessels. (A) Coronal reformatted image demonstrating a looped course of the common carotid artery (arrow). (B) Axial CT image demonstrating an additional vascular loop at the thoracic inlet (arrow). (C) Three-dimensional volume-rendered reconstruction demonstrating the spatial orientation and extent of tortuosity of the supra-aortic branch vessels.

Volume-rendered CTA also demonstrated that arterial tortuosity extended beyond the thorax, with an elongated and tortuous thoracoabdominal aorta (Fig. 4). No flow-limiting stenosis/occlusion, contrast extravasation, arterial thrombosis or vascular calcification was identified. Incidental calcified granulomas and fibrotic bands were noted in the right middle lobe and lingula. Overall, the imaging findings were radiologically suggestive of arterial tortuosity syndrome.

**Fig. 4:**
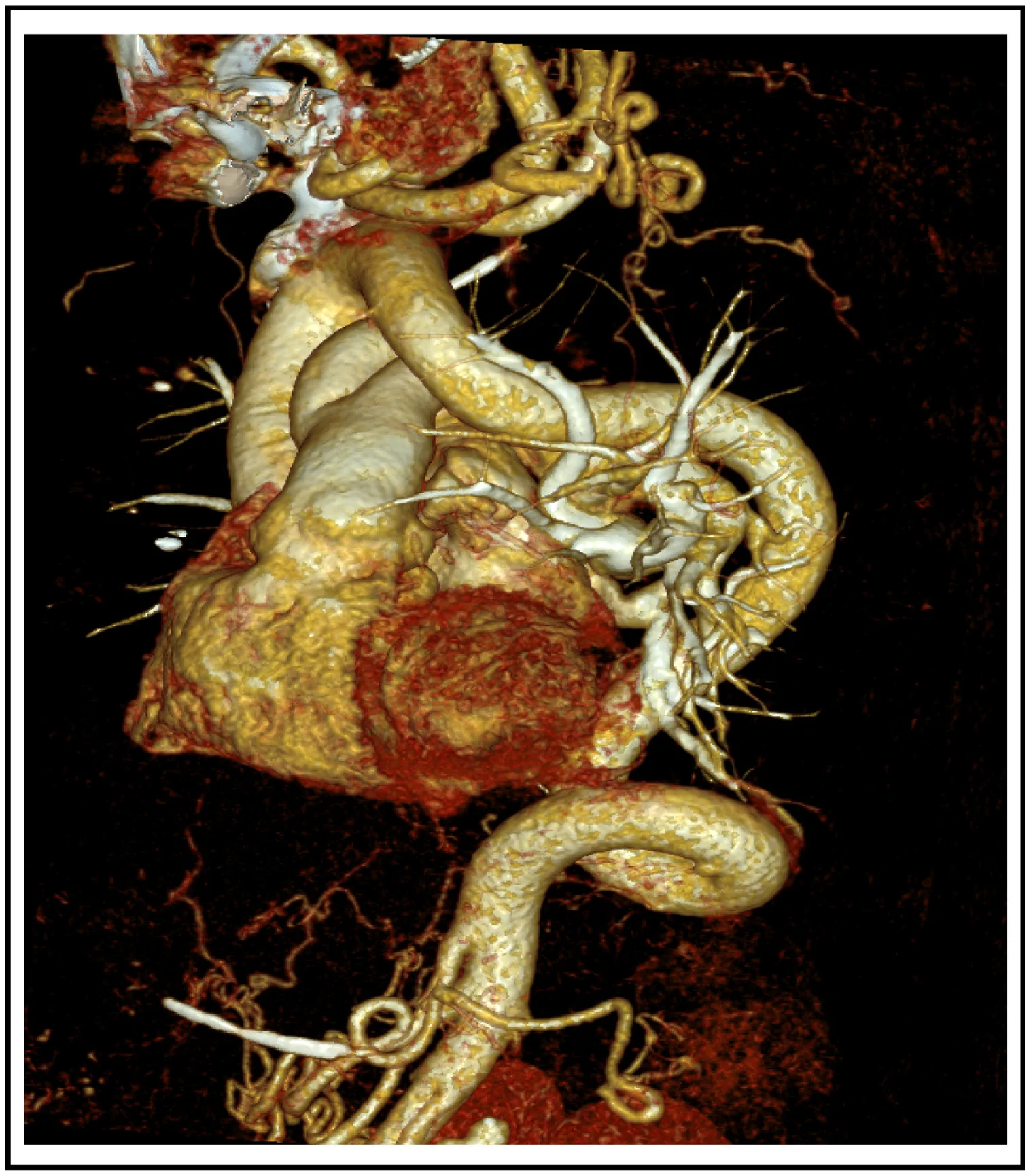
Long-segment volume-rendered CTA reconstruction demonstrating marked elongation and tortuosity of the thoracoabdominal aorta.

Based on the clinical presentation and characteristic imaging findings, the imaging findings were highly suggestive of arterial tortuosity syndrome with coronary artery involvement. Genetic confirmation was not done. Given the non-obstructive nature of the coronary stenosis and absence of acute vascular complications, the patient was managed conservatively with antiplatelet therapy, statins and antihypertensive medication aimed at vascular risk optimization and reduction of hemodynamic stress. At the one-month follow-up, she showed significant symptomatic improvement and was clinically doing well. She was counselled regarding the potential for future vascular complications and advised regular clinical and imaging follow-up for surveillance of aortic dimensions, coronary status and progression of arterial tortuosity.

## Discussion

ATS was first recognized as a distinct clinical entity by Ertugrul in 1967, who described a 10-year-old girl with widespread tortuosity and elongation of the major arteries, including the carotid arteries, aorta, and multiple visceral arteries, accompanied by fragmentation of the internal elastic membrane on histopathological examination and exclusion of acquired causes [12]. Subsequent genetic studies established ATS as an autosomal recessive connective-tissue arteriopathy caused by biallelic pathogenic variants in the *SLC2A10* gene on chromosome 20q13, which encodes the facilitative glucose transporter 10 (GLUT10) [13,14]. More than 20 pathogenic variants have been reported, resulting in defective or mislocalized GLUT10 protein. Although the precise transported substrate remains uncertain, both glucose and dehydroascorbic acid have been proposed as potential substrates, with GLUT10 localized to mitochondrial and endoplasmic reticulum membranes. Disruption of GLUT10 function has been associated with altered TGF-β signaling, increased oxidative stress, and extracellular matrix abnormalities that contribute to the vascular and connective-tissue manifestations of ATS [1,13,15]. An additional “vitamin C connection” hypothesis proposes that impaired intracellular handling of (dehydro) ascorbate may adversely affect collagen and elastin maturation and contribute to secondary TGF-β activation, providing a complementary explanation for the overlap between ATS and other connective-tissue disorders [16].

ATS is an exceptionally rare inherited arteriopathy, and its true incidence and prevalence remain uncertain because of its rarity and likely under-recognition, particularly among adults with incomplete syndromic manifestations. Most reported patients are diagnosed during childhood, but adult and late presentations have been increasingly recognized, suggesting that the disorder may remain undiagnosed when vascular tortuosity is attributed to more common connective-tissue or vascular conditions [1,3,[6], [7], [8],^10^]. The expanding recognition of adult cases is particularly relevant to radiologists because generalized arterial tortuosity may be the most conspicuous or even the initial clue to an underlying inherited arteriopathy.

Clinically, ATS has a broad spectrum, often presenting in infancy or childhood, but adult and “late” diagnoses are well recognized, particularly when connective-tissue features are subtle or attributed to more common syndromes [17]. Commonly affected vessels are large and medium-sized arteries that include the aorta, pulmonary arteries, and arch branches, but smaller arteries have also been seen [4]. The large Genet Med cohort/review by Beyens et al. showed that aortic and extra-aortic tortuosity are highly prevalent and that associated features such as inguinal/diaphragmatic hernia are common, supporting ATS as a multisystem disorder where vascular findings may coexist with apparently “non-specific” connective-tissue manifestations [1]. Affected vessels are prone to focal stenosis, aneurysm, dissection, and ischemic events. Reported cardiovascular complications include hypertension, stroke, and infarctions. Respiratory complications may arise due to vascular compression of airways and include difficult breathing and recurrent chest infections [17]. Our patient’s history of inguinal hernia repair and chronic polyarthralgia aligns with this broader phenotype, while the imaging pattern, diffuse tortuosity affecting the thoracic aorta, supra-aortic branches and abdominal branches, matches typical ATS distributions described in radiology-focused reports and reviews.

Imaging plays a central role in recognizing ATS because several reproducible radiological signs can raise suspicion for a syndromic arteriopathy. Reported CT findings include elongation and tortuosity of the aorta and its major branches, meandering vessels, clustered or looping arterial segments, and characteristic V-shaped or inverted-V configurations related to early pulmonary artery branching [6,10,17,18]. Tortuosity may appear as S- or C-shaped curves and can produce acute angulation known as kinking, which is graded as mild (≥ 60°), moderate (30°–60°), or severe (< 30°) [19]. In our case, the combination of early pulmonary trunk branching and diffuse multi-territory arterial tortuosity (including supra-aortic looping) provided a coherent radiological pattern supportive of ATS. The presence of these findings across multiple vascular territories is particularly important because an isolated tortuous vessel may be nonspecific, whereas widespread arterial involvement should prompt consideration of an inherited arteriopathy [1,3,10].

A key feature of this case is coronary involvement, which is reported less frequently than aortic or supra-aortic disease but is clinically important because it can drive symptoms and complicate invasive procedures. The present patient was presented in adulthood with exertional chest pain and dyspnea, and was found on CTA to have marked left main coronary tortuosity with focal kinking and mild to moderate luminal narrowing alongside diffuse systemic arterial elongation and tortuosity. A recent radiology “Images in Radiology” report highlights coronary tortuosity as part of the ATS imaging phenotype and also illustrates that clinically relevant vascular complications may occur in ATS [10]. Cardiovascular abnormalities are a major source of morbidity and mortality among patients with ATS due to aneurysm formation, arterial dissection, and ischemic events such as stroke and/or abdominal infarction [10].

Several previously reported adult ATS cases have demonstrated that the diagnosis may be established only after extensive vascular imaging, despite relatively subtle or nonspecific connective-tissue manifestations. Adult presentations have included diffuse arterial tortuosity identified during cardiovascular evaluation, cerebrovascular manifestations, and incidental recognition of the characteristic vascular phenotype [[6], [7], [8], [9],^17^]. In comparison, the present case is distinctive because the initial clinical presentation was exertional chest pain and dyspnea, leading to attempted conventional coronary angiography, during which severe systemic arterial tortuosity itself created a procedural challenge. Subsequent CT coronary angiography demonstrated marked left main coronary artery tortuosity with focal kinking, while systemic CTA simultaneously demonstrated extensive multi-territory arterial involvement. This combination illustrates how coronary symptoms may provide the initial clinical entry point to recognition of a previously unrecognized systemic arteriopathy.

In our patient, CT-CAG showed marked left main tortuosity with focal kinking and non-obstructive (40–45%) stenosis (CAD-RADS 2). Although coronary tortuosity and kinking may plausibly influence coronary flow dynamics and contribute to exertional symptoms, this relationship remains speculative in the present case because no functional ischemia testing was performed. While this degree of luminal narrowing is typically not flow-limiting at rest, coronary tortuosity and kinking can plausibly contribute to exertional symptoms by altering distal perfusion pressure and flow dynamics. Supporting this rationale, clinical literature outside ATS has associated severe coronary tortuosity with non-obstructive coronary disease presentations, particularly among women with angina-like symptoms [20]. Therefore, in ATS patients presenting with chest pain and low calcium score, coronary tortuosity/kinking should be considered a potential contributor to symptoms and a reason to individualize follow-up and risk-factor optimization. The decision for conservative management is consistent with the absence of dissection/aneurysm and the presence of non-obstructive coronary stenosis, while recognizing that ATS carries potential for vascular complications and therefore warrants structured follow-up rather than discharge from care.

The diagnosis of ATS may be challenging because arterial tortuosity can initially be interpreted as an isolated anatomical variant, age-related vascular change, or a manifestation of another inherited connective-tissue disorder. In patients presenting with chest pain, marked coronary tortuosity may also lead to an initial focus on conventional coronary artery disease, particularly when luminal narrowing is present. Invasive angiography can be technically difficult when generalized arterial tortuosity prevents catheter advancement or selective vessel engagement, as occurred in this patient. Recognition of diffuse tortuosity across multiple vascular territories should therefore prompt review of the entire available vascular tree rather than limiting assessment to the symptomatic vascular bed [1,6,7,10].

This patient's diagnosis was based primarily on the characteristic imaging phenotype rather than molecular confirmation. The diffuse involvement of multiple arterial territories, including the thoracic aorta, supra-aortic branches, abdominal branches, pulmonary arteries, and coronary arteries, strongly supported arterial tortuosity syndrome (ATS) [[1], [2], [3],^10^]. Differential considerations include other inherited connective-tissue arteriopathies, particularly Marfan syndrome, vascular Ehlers–Danlos syndrome (vEDS), and Loeys–Dietz syndrome (LDS), all of which may demonstrate arterial tortuosity or other vascular abnormalities. Marfan syndrome is typically characterized by aortic root dilatation together with skeletal, ocular, and other systemic features, whereas vEDS is associated with arterial and tissue fragility, easy bruising, thin translucent skin, and characteristic craniofacial features. LDS may show widespread arterial tortuosity and aneurysmal disease but is often accompanied by characteristic craniofacial, skeletal, and other systemic findings [1,2,10]. In the present case, no characteristic clinical features of these alternative syndromes were documented; however, because dedicated genetic and dysmorphology assessment was not performed, these conditions cannot be considered definitively excluded.

From a radiological perspective, recognition of ATS requires assessment beyond the symptomatic vascular territory. When marked tortuosity or elongation is identified, radiologists should evaluate the aorta, supra-aortic branches, pulmonary arteries, visceral vessels, and, when clinically appropriate, the coronary circulation for additional abnormalities. Multiplanar and three-dimensional CT reconstructions can facilitate recognition of vessel looping, kinking, elongation, and abnormal branching patterns. Identification of a diffuse multi-territory pattern should prompt consideration of an inherited arteriopathy and appropriate clinical or genetic evaluation. Early recognition is clinically relevant because ATS is associated not only with tortuosity but also with arterial stenosis, aneurysm, dissection, and other vascular complications [1,3,6,10].

Strengths of this case include multi-territory vascular phenotyping with CTA (multiplanar and 3D reconstructions) together with CT-CAG, allowing integrated description of systemic arterial tortuosity. Limitations include the absence of confirmatory genetic testing (preventing definitive genotypic confirmation and genotype–phenotype correlation), lack of functional ischemia tests, and limited longitudinal follow-up to assess progression or future complication risk.

### Learning points

1. Arterial tortuosity syndrome (ATS) should be considered in adults with diffuse, multi-territory arterial elongation and tortuosity, particularly when characteristic pulmonary artery branching abnormalities and supra-aortic looping are present.
2. Coronary artery involvement in ATS may manifest as marked tortuosity and focal kinking and can create substantial technical difficulty during conventional coronary angiography.
3. Multidetector CT angiography enables comprehensive vascular mapping, supports radiological recognition of ATS, identifies potentially important vascular complications, and facilitates surveillance when molecular confirmation is unavailable.

## Conclusion

This case highlights that ATS can present in adulthood with exertional chest pain and dyspnea. Coronary artery tortuosity with focal kinking and non-obstructive stenosis may simulate acute coronary syndrome, potentially leading to diagnostic confusion. Recognizing the characteristic imaging pattern of diffuse multi-territory arterial tortuosity with supportive pulmonary trunk branching and supra-aortic looping can enable diagnosis even when genetic testing is unavailable. Comprehensive vascular mapping should be considered in suspected ATS to define disease extent, identify complications, and inform follow-up, and coronary involvement should be actively assessed because it may influence symptom interpretation, procedural planning and follow-up strategy.

## Provenance and peer review

Not commissioned, externally peer-reviewed.

## Author contributions

PS: Conceptualization, data curation, supervision, literature review, writing - review & editing. SGC: Project administration, literature search and review, writing - original draft, writing - review & editing. AS: Data curation, literature review, writing - review & editing. NKS: Radiological interpretation, validation, writing - review & editing. AM: Clinical supervision, methodology, validation, writing - review & editing. SKS: Clinical supervision, methodology, validation, writing - review & editing. All authors read and approved the final manuscript.

## Data availability statement

All relevant data supporting this case report are included within the article.

## Patient consent

Written informed consent was obtained from the patient for publication of this case report and the accompanying clinical information and imaging studies. A copy of the written consent is available for review by the Editor-in-Chief of *Radiology Case Reports* upon request. All identifying personal information has been removed to protect the patient's privacy and confidentiality.

## References

[1] Beyens A., Albuisson J., Boel A., Al-Essa M., Al-Manea W., Bonnet D. (2018). Arterial tortuosity syndrome: 40 new families and literature review. Genet Med.

[2] Callewaert B.L., Willaert A., Kerstjens-Frederikse W.S., De Backer J., Devriendt K., Albrecht B. (2008). Arterial tortuosity syndrome: clinical and molecular findings in 12 newly identified families. Hum Mutat.

[3] Esmel-Vilomara R., Valenzuela I., Riaza L., Rodriguez-Santiago B., Roses-Noguer F., Boronat S. (2023). Arterial tortuosity syndrome: phenotypic features and cardiovascular manifestations in 4 newly identified patients. Eur J Med Genet.

[4] Naunheim M.R., Walcott B.P., Nahed B.V., MacRae C.A., Levinson J.R., Ogilvy C.S. (2011). Arterial tortuosity syndrome with multiple intracranial aneurysms: a case report. Arch Neurol.

[5] Rodríguez-Capitán J., Macías-Benítez M., Conejo-Muñoz L., Cordero-Aguilar A., López-Salguero R., Pérez-Villardón B. (2020). Arterial tortuosity syndrome: a late and unexpected diagnosis and description of a novel likely pathogenic mutation. Rev Esp Cardiol (Engl Ed).

[6] Elnaggar M.E., Aly M.M., Abduljawad H., Bubshait M., Ebrahim W.H. (2022). A rare case of arterial tortuosity syndrome in an adult. Radiol Case Rep.

[7] Mohan S., Kota P.B., George A.P., Keshava S.N. (2020). Arteries gone astray: adult presentation of arterial tortuosity syndrome. Indian J Vasc Endovasc Surg.

[8] Castori M., Ritelli M., Zoppi N., Molisso L., Chiarelli N., Zaccagna F. (2012). Adult presentation of arterial tortuosity syndrome in a 51-year-old woman with a novel homozygous c.1411+1G>A mutation in the SLC2A10 gene. Am J Med Genet A.

[9] Cotti Piccinelli S., Premi E., Bonacina S., Gilberti N., Vergani V., Gamba M. (2021). Arterial tortuosity syndrome causing recurrent transient ischemic attacks in young adult: a case report. BMC Neurol.

[10] Van Berkel B., Sneyers V. (2024). Arterial tortuosity syndrome. Radiology.

[11] Gagnier J.J., Kienle G., Altman D.G., Moher D., Sox H., Riley D.S. (2013). The CARE guidelines: consensus-based clinical case reporting guideline development. J Med Case Rep.

[12] Ertugrul A. (1967). Diffuse tortuosity and lengthening of the arteries. Circulation.

[13] Zaidi S.H., Peltekova V., Meyer S., Lindinger A., Paterson A.D., Tsui L.C. (2005). A family exhibiting arterial tortuosity syndrome displays homozygosity for markers in the arterial tortuosity locus at chromosome 20q13. Clin Genet.

[14] Coucke P.J., Willaert A., Wessels M.W., Callewaert B., Zoppi N., De Backer J. (2006). Mutations in the facilitative glucose transporter GLUT10 alter angiogenesis and cause arterial tortuosity syndrome. Nat Genet.

[15] Zoppi N., Chiarelli N., Cinquina V., Ritelli M., Colombi M. (2015). GLUT10 deficiency leads to oxidative stress and non-canonical αvβ3 integrin-mediated tgfβ signalling associated with extracellular matrix disarray in arterial tortuosity syndrome skin fibroblasts. Hum Mol Genet.

[16] Segade F. (2010). Glucose transporter 10 and arterial tortuosity syndrome: the vitamin C connection. FEBS Lett.

[17] Kumar P. (2021). Arterial tortuosity syndrome: a case report and literature review. Cardiovasc Imaging Asia.

[18] Bhat V., Al Muzrakchi A. (2008). Meandering vessels: a sign of arterial tortuosity on plain chest radiography. Heart Views.

[19] Weibel J., Fields W.S. (1965). Tortuosity, coiling, and kinking of the internal carotid artery. II. Relationship of morphological variation to cerebrovascular insufficiency. Neurology.

[20] Zebic Mihic P., Saric S., Bilic Curcic I., Mihaljevic I., Juric I. (2023). The association of severe coronary tortuosity and non-obstructive coronary artery disease. Medicina (Kaunas).

